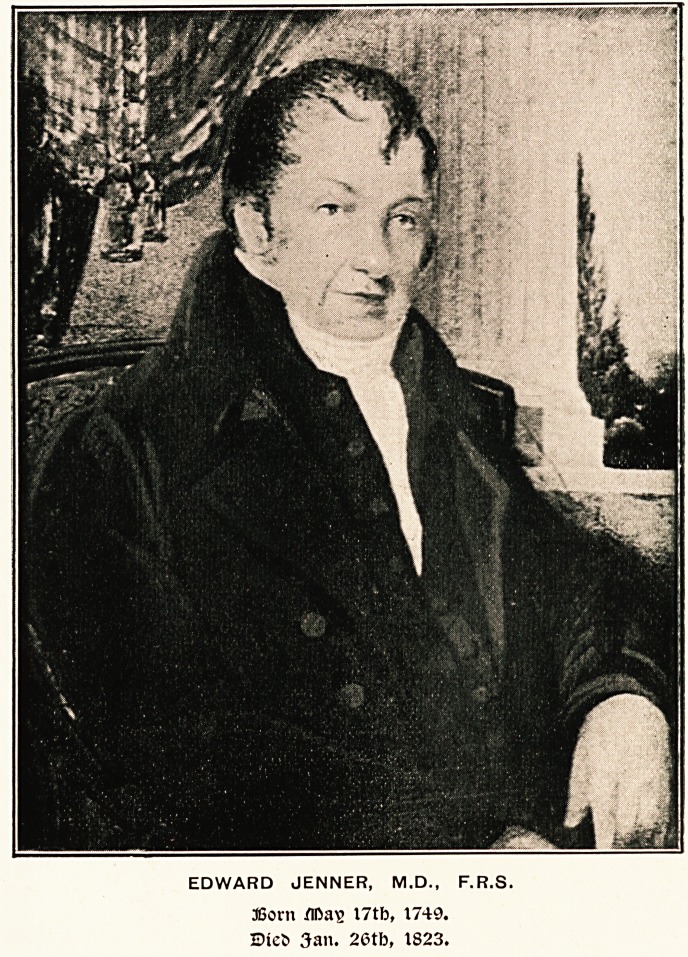# Jenner

**Published:** 1896-09

**Authors:** 


### JENNER.

There is no need here to enter into the details of the early work in connection with vaccination; for in this centenary of its discovery Jenner's praises have been sung and his beneficent work has been celebrated with much pomp and circumstance in many journals.[1] But as it would not be quite in accordance with the fitness of things that a medical periodical published in his own county should allow the occasion to pass unnoticed, we are glad to be able to reproduce a portrait of him which has not been previously issued. The picture on the opposite page is the exact size of a water colour now in the possession of the family of the late Mr. William Smith, formerly in practice in Pembroke Road, Clifton. Its history is interesting. A Society with which Jenner had been connected was desirous of having his portrait, for which he sat to a local artist, whose name, owing to the unfortunate loss of documents, is not known. In course of time the picture became by purchase the property of Mr. George Smith, the father of Mr. William Smith and grandfather of Mr. Munro Smith, to whom we are indebted for the obtaining of the picture. Mr. George Smith, who was at the time living at Berkeley, and was a personal friend of Jenner's, considered it a very accurate likeness. In the original picture the face is fresh-coloured, the eyes are blue, and the hair is grey.

[1] Reference should be made to *Practitioner*, May; *Münchener medicinische Wochenschrift*, May 12; *Nederlandsch Tijdschrift voor Geneeskunde*, May 16 *Australasian Medical Gazette*, May 20; *Boletin del Consejo superior de Salubridad* May 22; *British Medical Journal*, May 23; *Gazette médicale d'Orient*, August 15

## Figures and Tables

**Figure f1:**